# Effect of muscarinic blockade on the speed of attention shifting, read-out delays and learning

**DOI:** 10.1007/s00213-025-06757-3

**Published:** 2025-02-15

**Authors:** Alexander Thiele, Agnes McDonald Milner, Corwyn Hall, Lucy Mayhew, Anthony Carter, Sidharth Sanjeev

**Affiliations:** 1 Biosciences Institute, Newcastle University, Framlington Place, Newcastle upon Tyne, NE2 4HH UK; 2 School of Psychology, Newcastle University, Framlington Place, Newcastle upon Tyne, NE2 4HH UK

**Keywords:** Attention, Acetylcholine, Learning

## Abstract

The study aimed to investigate to what extent blockade of muscarinic receptors affects the speed of endogenous versus exogenous attentional shift times, and how it affects learning of attention shifting, cue detection and signal readout. Subjects viewed an array of 10 moving clocks and reported the time a clock indicated when cued. Target clocks were indicated by peripheral or central cues, including conditions of pre-cuing. For peripheral and central cuing, it yielded a compound measure of how long it took to detect the cue, shift attention to the relevant clock and read the time on the clock. For the pre-cue condition it yielded a measure of how long it took to detect the cue and read the time on the clock when attention could have been pre-allocated to the target clock. In study 1, each subject participated in 2 sessions (scopolamine/placebo), whereby the order of drug intake was counterbalanced across subjects, and subjects were blinded to conditions. Scopolamine/placebo was administered before a psychophysical experiment was conducted. In study 2, the effect of muscarinic blockade on learning induced improvements of cue detection, attention shift times (for exogenous and endogenous conditions), and signal readout was investigated. Here scopolamine/placebo was administered immediately after the first (of two) psychophysical sessions, whereby a given subject either received scopolamine or placebo pills. Confirming previous results, we show that pre-cuing resulted in the shortest read-out delays, followed by exogenous cuing, with endogenous read-out delays being slowest. Scopolamine application increased readout-delays in a dose dependent manner. This was mainly driven by increased readout-delays for pre-cue conditions, and to some extent for exogenous cue conditions. It suggests that muscarinic blockade affected the ability to pre-allocated attention to a cued location, as well as to react to peripheral cues. Additionally, blockade of muscarinic receptors immediately after the first session reduced learning dependent improvement of read-out delays. These results demonstrate that muscarinic receptors play an important in detecting cues, and fast read-out of cued information, and they contribute to the learning thereof.

**Supplementary Information:**

The online version contains supplementary material available at 10.1007/s00213-025-06757-3.

## Introduction

The speed with which covert attention can be shifted between different locations, has been investigated using many different paradigms, dating back to experiments performed by Wilhelm Wundt using a complication clock apparatus (Wundt 1883). Orienting paradigms (Posner 1980; Yantis and Jonides 1990; Carlson et al. 2006), paradigms investigating attentional gating (Reeves and Sperling 1986) and visual search paradigms (Wolfe et al. 2000) have been used in different forms. These studies have demonstrated that two types of attention can be dissociated, namely bottom-up and top-down attention (Posner 1980; Duncan 1984; Posner and Petersen 1990; Theeuwes 1991; Carlson et al. 2006). Bottom-up attention is reflexive and triggered by salient unexpected stimuli in the external world (often peripheral cues in experimental situations), conversely top-down attention is wilful, and can be triggered by central cues (which often need to be interpreted in experimental situations). These two types of attention also differ in terms of how quickly they can be allocated (shifted) to stimuli of interest. Bottom-up attention shifts are faster than top-down attention shifts by ~ 50-100ms (Posner 1980; Muller and Rabbitt 1989; Carlson et al. 2006; Chakravarthi and VanRullen 2011).

Attentional selection is assumed to be driven by frontal and parietal cortex (Corbetta and Shulman 2002; Moore and Armstrong 2003). Parietal and frontal regions influence sensory processing directly via feedback, but also indirectly, via connections to cholinergic neurons in the basal forebrain that have ascending projections to sensory areas (Russchen et al. 1985; Sarter et al. 2005). Critically, ample evidence for the involvement of acetylcholine (ACh) in attentional modulation exists (Nelson et al. 2005; Robbins 2005; Sarter et al. 2005; Parikh et al. 2007; Furey et al. 2008). Disrupting cholinergic fibres originating in the basal forebrain, thereby depleting cortical areas of ACh, results in attentional impairments (McGaughy et al. 2002; Sarter et al. 2005). Attentional deficits in Alzheimer’s disease partly arise from cholinergic dysfunction (Nobili and Sannita 1997). Increasing acetylcholine availability through application of acetylcholine-esterase inhibitors in human subjects improved the benefits of top-down (but not bottom up) attention on reaction times (Rokem et al. 2010). Infusion of the muscarinic blocker scopolamine into macaque parietal cortex results in a dose-dependent increase in reaction times and a decrease in accuracy, when locations were exogenously cued, with the biggest effect for validly cued locations (Davidson et al. 1999; Davidson and Marrocco 2000). Muscarinic blockade also reduced performance in sustained visual attention task, which can be conceptualized as an endogenous attention task (Ellis et al. 2006), and reduces attentional modulation of cell responses in primary visual cortex and frontal eye field neurons in macaque monkeys under endogenously cued covert attention conditions (Herrero et al. 2008; Dasilva et al. 2019). These studies suggest that endogenous and exogenous attention are both dependent on muscarinic signalling, but a direct test has, to the best of our knowledge, never been done.

To test the role of muscarinic signalling in exogenously, endogenously, and pre-cued attention shifts and read-out delays, we used a variant of Wundt’s complication-clock apparatus (Wundt in Carlson et al. 2006). It involves participants watching a fixation point surrounded by moving clocks. After a clock is cued, participants shift their attention to that clock and record the position of the hand they perceived on the clock when the clock was cued. This provides a compound measure of how long it takes to detect the cue, shift attention, and read the time on the clock for the exogenous and endogenous cue conditions. Assuming that attention is pre-allocated to the relevant clock in the pre-cue condition, it provides a measure of how long it takes to detect the cue and read the time on the clock. We refer to these measures as ‘readout-delay’ irrespective of condition, noting that they may entail different numbers and types of component processes. In study 1 participants performed the task twice on different days, either after having taken oral doses of scopolamine, or placebo pills.

Muscarinic receptors also play an important role in neuronal plasticity (McKenna et al. 1989; Metherate and Weinberger 1990; Weinberger and Bakin 1998; Barros et al. 2002) and learning (McGurk et al. 1988, 1991; Carli et al. 1997; Izquierdo et al. 1998; Thiel et al. 2002; Hasselmo 2006; Barker and Warburton 2009; Thiele 2013). A recent study has shown that attention shift times are affected by learning (*Thiele et al.*,* Biorxiv*). We therefore also investigated the role of muscarinic signalling in the learning dependent improvement of attention shift times and read-out delays. Here an oral dose of either placebo or scopolamine was given to participants after the first experimental session, and they then performed a second experimental session on the following day.

We found that muscarinic blockade affected readout-delay in a dose dependent manner. When a high dose was given muscarinic blockade mostly increased readout-delays for the pre-cue condition, followed by the exogenous condition, with no significant effects on the endogenous condition. Moreover, application of the muscarinic blocker scopolamine during the immediate consolidation period resulted in reduced improvements of readout-delays.

## Methods

### Participants

Study 1: Eighty-one participants took part in the experiment, where the effect of muscarinic blockade on readout-delays was investigated. This sample consisted of 34 males and 47 females, with an age range of 18–29 years, median 22.

Study 2: Twenty subjects took part in the experiment where the effect of muscarinic blockade on learning induced improvements of readout-delays was tested. The sample consisted of 20 students (10 male/10 female) from Newcastle University. Participants were selected based on opportunity sampling and had an age range of 19–22 years old, median 21.

All participants had normal or corrected-to-normal vision. They were required to sign a consent form to participate in the experiment. The consent form gave information about the task, the detailed procedures, the purpose of the study, how data would be used and anonymized. Here they also confirmed that they had not eaten for 2 h before the experiment or drunk alcohol for 12 h before the experiment, were not hungover at the day of the experiment and had abstained from using recreational drugs for > 48 h. Subjects were only included if they were at least 18 years old, not allergic to Kwell pills (active ingredient hyoscine hydrobromide, also known and referred to as scopolamine) or if they were taking any medication which should not be taken alongside Kwell pills (supplementary materials). High blood pressure and pregnancy were also exclusion criteria. They were instructed not to drive or operate any motorised equipment for up to 6 h after a session. Ethics were approved by the Faculty of Medical Sciences ethics committee at Newcastle University (01016_1/2015 to 01016_6/2023). Subjects had to perform the task twice, whereby each session had to be performed on separate days (at about the same time of a day, i.e. if a subject did session one in the morning, they performed session 2 on a different day, but also in the morning).

### Design

Two separate designs were used for the Study 1 and Study 2.

Study1: Assessing the effects of scopolamine on readout-delays. The design of the experiment was within-subject repeated measurements. Each participant carried out the experimental and placebo conditions in separate sessions. The two sessions were performed on different days, but at roughly the same time of day to control for effects of circadian rhythm on attention. They were informed prior to the experiments that in one of the two sessions they would receive either a dose of Kwell pills, or a dose of placebo pills. Subjects were given the respective pills 45 min before the start of the experiments, after which they were free to spend the next 45 min in whichever way they chose. Forty-one subjects were given a dosage of 300 mg scopolamine (these subjects received one Kwell pill, when the ‘drug’ session was selected), and forty subjects were given a dosage of 600 mg scopolamine (these subjects received two Kwell pills, when the ‘drug’ session was selected). Placebos were homeopathic arnica 6 C. Forty participants performed the experimental condition first and the remaining forty-one performed the placebo condition first, to counterbalance potential order (learning) effects.

Study 2: Assessing the effects of scopolamine on learning induced improvement of readout-delays: The design of the experiment was between-subject repeated measurements. Each participant carried out two separate sessions, whereby one group of subjects received two Kwell pills immediately after the first session, and the other group received the placebo pills immediately after the first session. A single dosage of 600 mg was used in the subjects that received the drug treatment. Placebo treatment consisted of two unflavoured chewable glucose pills. Subjects were blinded to the condition. There was no scientific rationale for using a different placebo in study 2. They were then free to leave the premises, to return at around the same time on the following day, when they performed the second session.

An alternative design could have been where some subjects performed both sessions with placebo (placebo-placebo) and compare this with placebo-drug and drug-placebo subject groups to delineate the effects of muscarinic blockade on inter-session learning. However, this would always entail a comparison against a group of subjects that were affected by muscarinic blockade in one of the sessions. We could also have added a group of subjects that took scopolamine in both sessions. But then muscarinic blockade could interfere with encoding (if we had a ‘drug-drug’ treatment group) in the first session. Also, there could be habituation effects if subjects take the pills twice in succession, which could negatively interact with the blocked learning. For these reasons we decided on the stated design, where neither of the subject groups were affected by muscarinic blockade while performing the task.

### Procedure

Before the experiment, participants were given the experiment information sheet and questionnaire via email. They had to fill out the pre-experiment questionnaire and email it to the experimenter. Upon arrival for the first session, they were given a consent form to read and sign. They were briefed again about the experiment, i.e. the psychophysical task in conjunction with muscarinic blockade. Any questions about the task were then answered. This was followed by a 30-trial practice session, where they familiarized themselves with task and obtained initial training.

**Fig. 1:**
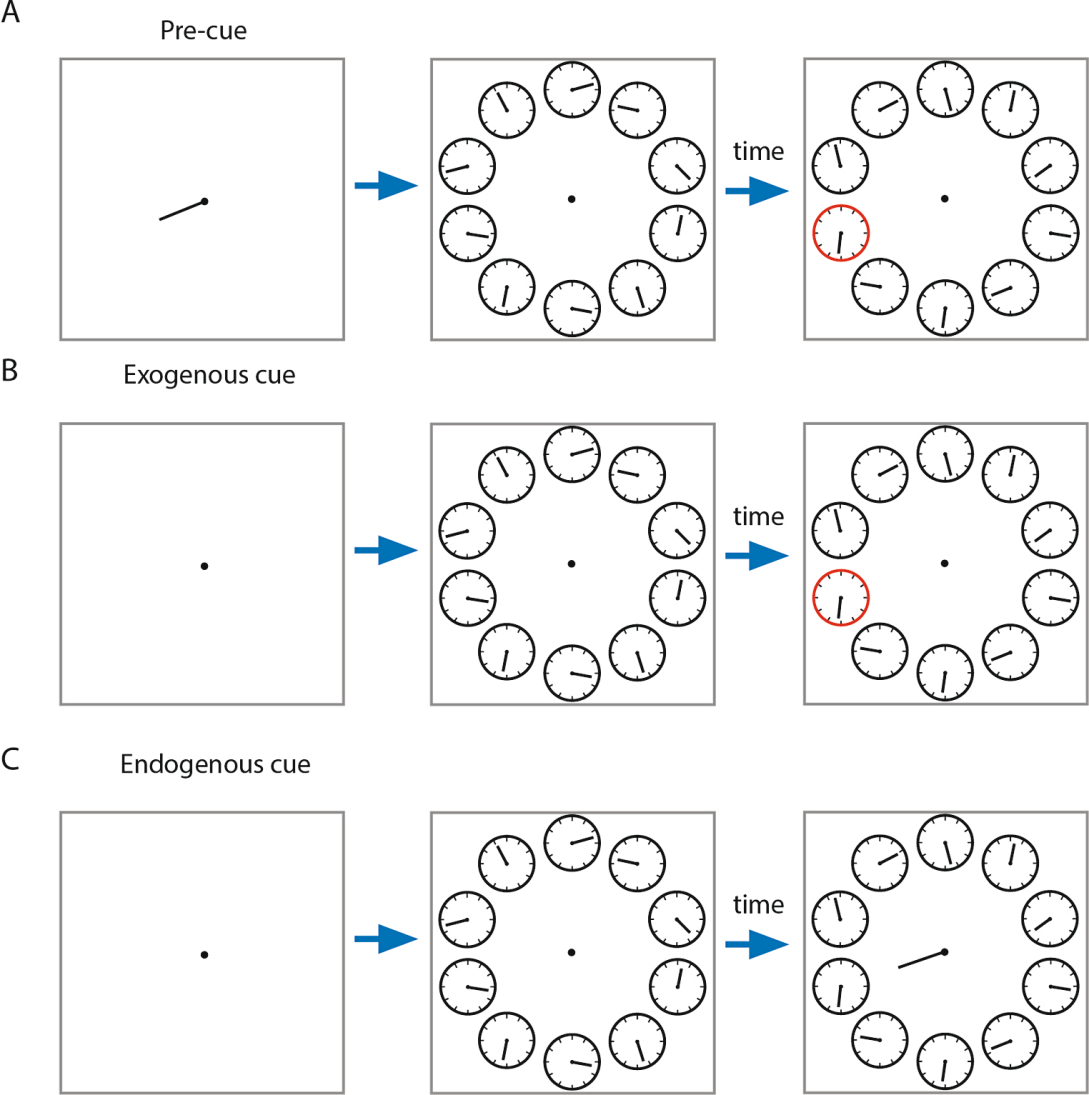
Experimental setup for the 3 attention conditions. **A**) The top row indicates the events that occur in the pre-cue condition, **B**) middle row the events in the exogenous condition, and **C**) bottom row the events that occur in the endogenous cuing condition. The blue arrow indicates time passing. This was fixed for the first period, but variable for the second period

The psychophysical task was as described in (Carlson et al. 2006). It consisted of 3 different attention conditions (pre-cue, exogenous and endogenous cuing), where subjects had to read the time on one of 10 possible clocks, when the relevant clock was cued (see below). Each condition occurred 50 times, randomly selected without replacement. Each trial started with a central fixation spot, which subjects were asked to fixate. Before the start of the trial the computer randomly assigned which clock would be cued on that given trial. In the pre-cue conditions the fixation spot appeared along with a central cue, a short line (length: 2 deg of visual angle (dva), emanating from close to the fixation cross, pointing to the future relevant clock location. The cue was presented for 5 frames (50ms) after which it disappeared while the fixation spot stayed on. After 1 s, 10 clock faces (~ 2.5 dva diameter) appeared simultaneously at an eccentricity of 7 dva on the screen. Each clock showed a single clock hand with a randomly allocated starting position (see Fig. 1 for an example). The clock hands rotated at 1 Hz, i.e. one revolution per second. At a randomly selected time after the start of the trial (drawn from a uniform distribution from 100-1000ms after clock onset, steps of 10ms) one of the clocks would be cued (details below). Shortly after the cue (1000ms), the clocks were replaced by one central clock. Participants were required to indicate the position of the hand on the clock when it was cued by moving the hand of the second clock to the same position using the left/right arrow buttons of the keyboard. There were 3 different conditions in the task; (1) in the exogenous cue condition, the rim of the cued clock changed from black to red at the time of cuing for 5 frames (50ms). (2) In the endogenous cue condition, a line appeared emanating from the fixation point pointing towards one of the clocks (for 5 frames, i.e. 50 ms). (3) In the baseline (pre-cue) condition, a line appeared before the clock faces (as described above), indicating the position of the clock that would be cued. That specific clock was then cued during the trial as in the exogenous cuing condition. Figure 1 illustrates the progression of events in each of the 3 conditions (see also the videos in Carlson et al. 2006).

### Apparatus/ materials

The experiment was run on windows PC running MATLAB 2015a (62-bit) (The MathWorks, Inc.) using PsychToolbox extentions (Brainard 1997). The stimuli were displayed on an Iiyama 22” monitor (100 Hz, 2,048 × 1536 resolution) that was controlled by the PC. Observers sat with their chin on a chin rest 60 cm away from the monitor. Responses were collected from the keyboard, using the left and right arrow keys to move the clock hand and the space bar to confirm the participant’s observed time.

### Data analysis

The latency between the real time of cueing and the time of cueing reported/perceived by the participant (i.e. the time indicated by the adjusted clock hand) was calculated for each trial. This could result in latencies of 0-999ms (one full revolution of the clock hand) in principle. However, latencies that exceeded 900 ms were calculated as latency-1000. This cutoff latency was accepted as it allows for some random perceptual/memory errors to be distributed around the actual time that was present at cuing. It resulted in all final latencies to range from − 99ms to 900ms. The mean latency for the 3 cuing and the 2 experimental (drug/no drug) conditions were then calculated for each participant.

To investigate whether scopolamine affected readout-delays a mixed model ANOVA was used. This was based on subject means calculated for the three cuing conditions (3 cuing conditions, within subject repetition), experimental condition (drug/no drug, within subject repetition), drug dosage (300 mg/600 mg, between subject factor), or the order of the experimental condition (scopolamine or placebo in first session, between subject factor).

To investigate whether scopolamine affected the learning of readout-delays a mixed model ANOVA was also used, but with different factors. This was based on subject means calculated for the three cuing conditions (3 cuing conditions, within subject repetition), and the experimental condition (drug/no drug, between subject factor). Given the repeated measures nature of our analyses between-subject variance was removed before calculating S.E.M for all analyses. This was done by subtracting each subject means (across the 3 attention and 2 treatment conditions), from the respective condition means.

## Results

We will first describe the results regarding the effects of muscarinic blockade on readout-delays, followed by describing the effects on learning induced improvements of readout-delays.

### Effects of muscarinic blockade on read-out delays

**Fig. 2:**
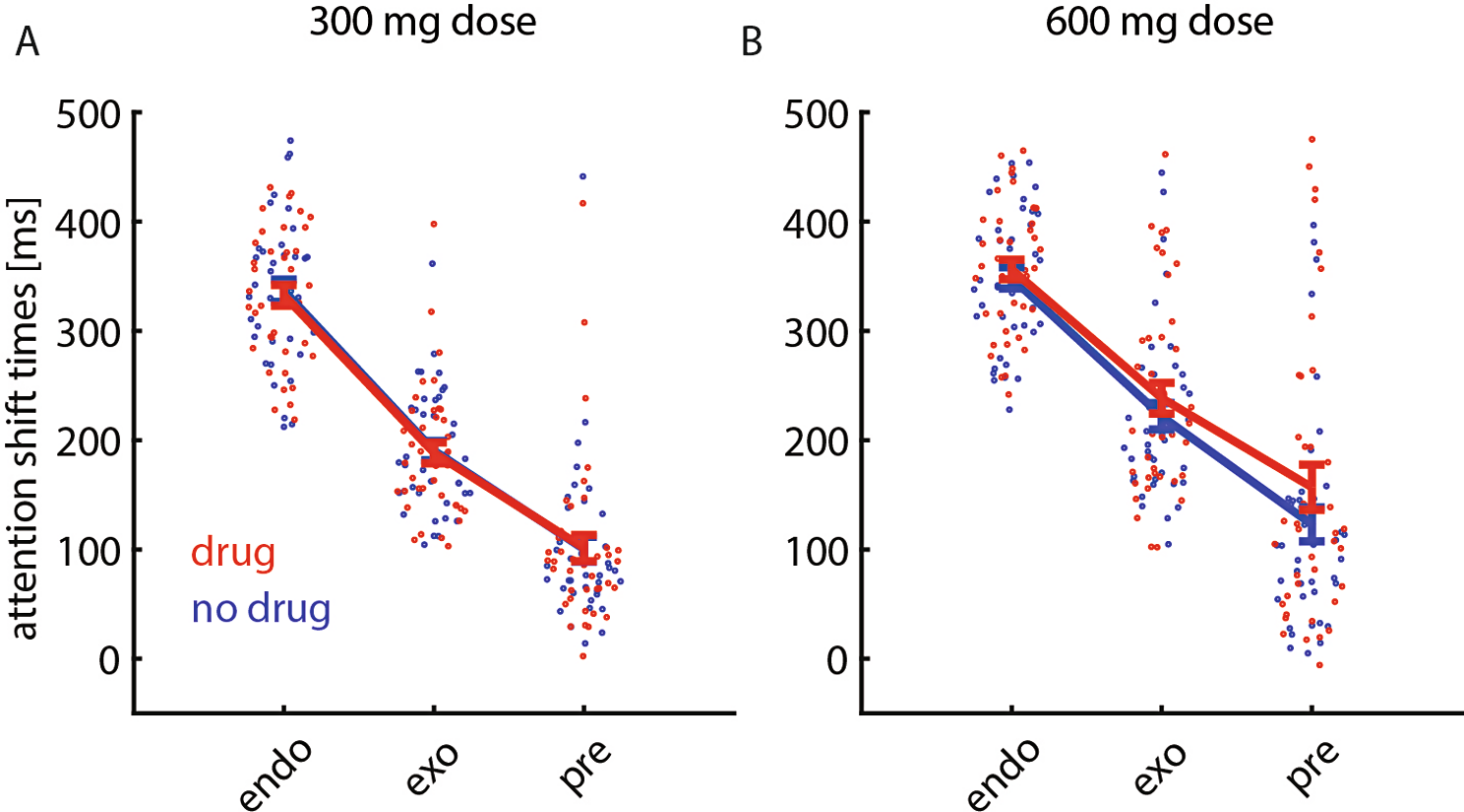
Readout-delays for the 3 attention conditions when subjects were not under the influence of scopolamine (blue) and when they were under the influence of scopolamine (red). Cuing conditions are indicated along the x-axis, mean readout delays along with S.E.M are indicated along the y-axis. (**A**) data for subjects in the 300 mg drug study (*n* = 41), (**B**) data for subjects in the 600 mg drug study (*n* = 40)

Eighty-one participants performed the study, whereby 41 participants took the placebo before the first session, and 40 participants took the Kwell pill(s) before the first session (order of experimental conditions). Forty-one subjects took a dose of 300 mg in the drug condition (21 subjects took the placebo first, the remaining subjects took the drug first), and the remaining 40 subjects took a dose of 600 mg in the drug condition (20 subjects took the placebo first, the remaining subjects took the drug first). Confirming previous results (Carlson et al. 2006), we found that readout-delays were shortest in the pre-cue condition, followed by the exogenous cuing, and longest in the endogenous cuing condition (Fig. 2).

The mixed model ANOVA revealed that there was a significant main effect of cue condition, a significant main effect of drug condition, a significant effect of drug level (Table 1). Additionally, there were significant interactions between order and drug (y/n), as well as between drug (y/n) and drug-level (300/600 mg) (Table 1).

**Table 1:** Mixed model ANOVA details. *Factor* indicates the parameter of interest, *df* shows the degrees of freedom, *F* and *p* give the F- and p-values respectively. * symbol denotes interaction between factors

Factor	df	F	*p*
Intercept (subject effects)	(1,462)	1130.8	< 0.001
cue-type	(2,462)	784.7	< 0.001
drug (y/n)	(1,462)	4.1	0.044
order	(1,462)	2.7	0.099
drug-level (300/600 mg)	(1,462)	7.0	0.008
order*drug (y/n)	(1,462)	11.6	< 0.001
order*cue-type	(2,462)	0.9	0.399
drug (y/n) * cue-type	(2,462)	1.0	0.363
order*drug-level (300/600 mg)	(1,462)	0.1	0.819
drug (y/n) *drug-level (300/600 mg)	(1,462)	6.5	0.011
cue-type*drug-level (300/600 mg)	(2,462)	2.3	0.102
order*drug(y/n) *cue-type	(2,462)	0.0	0.954
order*drug(y/n) *drug-level (300/600 mg)	(1,462)	0.1	0.793
order*cue-type*drug-level (300/600 mg)	(2,462)	1.7	0.192
drug(y/n) *cue-type*drug-level (300/600 mg)	(2,462)	0.7	0.514
order*drug(y/n) *cue-type*drug-level (300/600 mg)	(2,462)	0.6	0.553

Scopolamine intake increased readout-delays for all 3 cue types, with no interaction effect between drug (y/n) and cue types (Table 1, and Fig. 2). Drug level significantly affected readout-delays, whereby visual inspection (Fig. 2) suggests that 300 mg hardly resulted in a slowing, while 600 mg caused larger slowing. This impression is corroborated by analysing drug-levels separately. The 300 mg dose did not affect readout-delays (main effect of drug: *p* = 0.689, F(1,234) = 0.2, mixed model ANOVA), while the 600 mg dose significantly affected readout-delays (main effect of drug: *p* = 0.004, F(1,228) = 8.7, mixed model ANOVA).

**Fig. 3:**
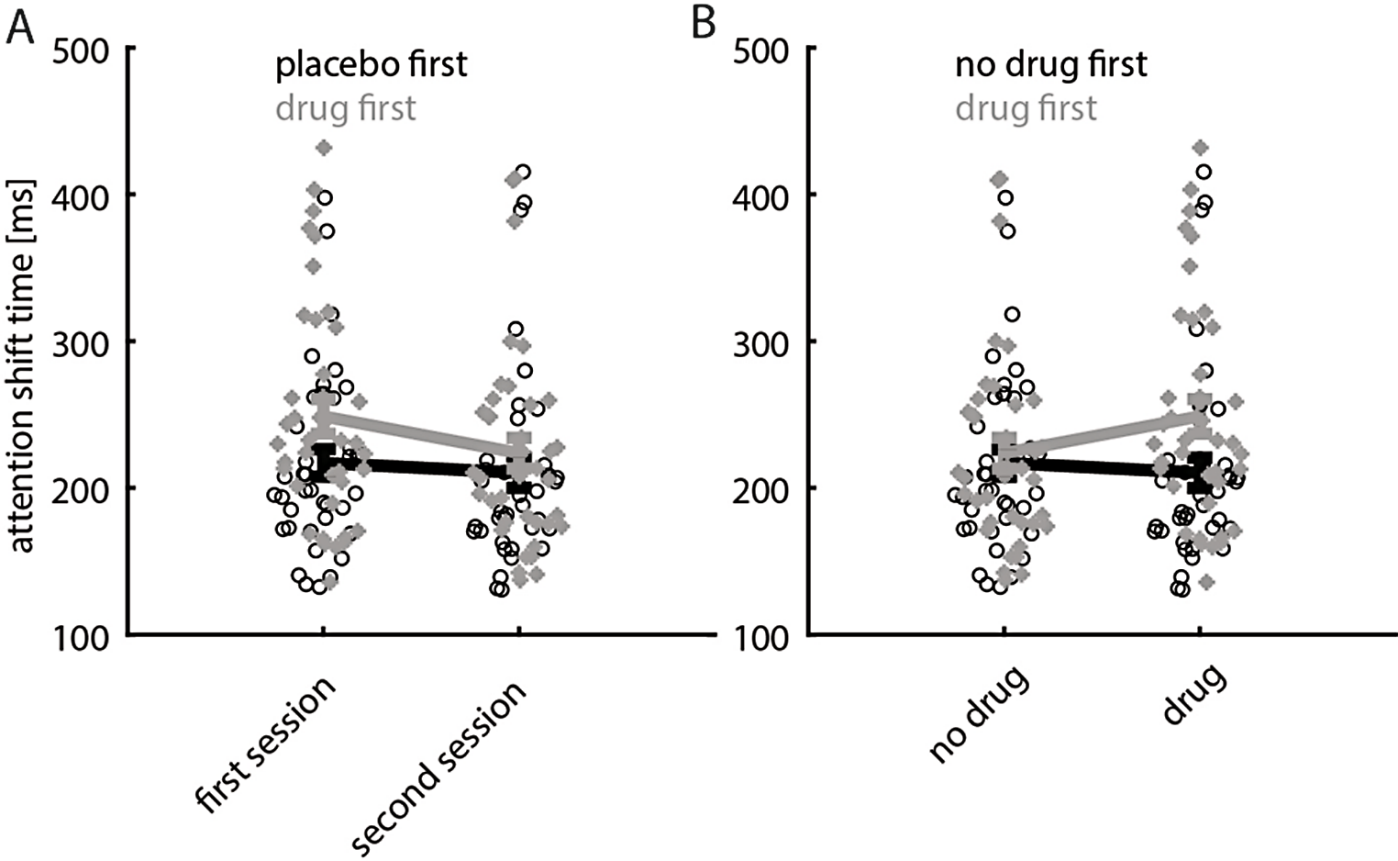
Average readout-delays (across the 3 conditions). **A**) Readout-delays as a function of session (first, second) and as a function of whether they were under the influence of scopolamine (drug first, grey) in the first session. **B**) Readout-delays as a function of whether they were under the influence of scopolamine or not, separated whether scopolamine was given in the first session (grey, drug first) or whether placebo was given in the first session. Note that for grey data points in B that are located on the right correspond to participants who took the drug in the first session, while grey data points on the left correspond to the same subjects who participated under placebo conditions (hence the no drug label) in the second session. Session type is indicated along the x-axis, mean readout-delays along with S.E.M are indicated along the y-axis

The order of placebo/drug sessions itself was not significant (*p* = 0.099, mixed model ANOVA, Table 1) which might indicate that readout-delays did not differ between the first and the second session. However, there was a significant interaction between drug (y/n) and order (*p* < 0.001, Table 1). Drug application generally increased readout-delays, and hence any learning (improvement) between session, might be concealed in the group that was under the influence in the second session. This argument is supported by the significance of the drug* order interaction (mixed model ANOVA: *p* < 0.001, Table 1). Subjects had shorter readout-delays in the second compared to the first experimental session, provided they took the drug in the first session (Fig. 3).

Based on Fig. 2 it appears that readout-delays were most strongly increased by muscarinic blockade in the pre-cue condition, when subjects were exposed to a 600 mg dose. To investigate this further we performed post-hoc analyses on the drug effects for the three cue types separately. This showed that read-out delays were significantly increased when the drug was taken for the pre-cue condition (Table 2, significant main effects of both drug and drug-level), there was a significant main effect of drug level for the exogenous cuing condition, with no main effects of drug or drug-level for the endogenous cuing condition.

**Table 2:** Mixed model ANOVA for the three cuing conditions. *Factor* indicates the parameter of interest, *df* shows the degrees of freedom, *F* and *p* give the F- and p-values respectively. * symbol denotes interaction between factors

Factor	F	DF1	DF2	*p*
**Endogenous cuing**				
order	1.59	1	154	0.208
drug (y/n)	0.21	1	154	0.641
drug-level (300/600 mg)	3.13	1	154	0.078
order* drug (y/n)	11.39	1	154	< 0.001
order*drug-level (300/600 mg)	0.82	1	154	0.365
drug (y/n) * drug-level (300/600 mg)	1.73	1	154	0.189
order* drug (y/n) * drug-level (300/600 mg)	1.30	1	154	0.255
**Pre-cuing**				
order	2.42	1	154	0.121
drug (y/n)	5.24	1	154	0.023
drug-level (300/600 mg)	4.08	1	154	0.044
order* drug (y/n)	3.20	1	154	0.075
order*drug-level (300/600 mg)	0.18	1	154	0.664
drug (y/n) * drug-level (300/600 mg)	5.72	1	154	0.017
order* drug (y/n) * drug-level (300/600 mg)	0.55	1	154	0.457
**Exogenous cuing**				
order	2.04	1	154	0.154
drug (y/n)	1.77	1	154	0.184
drug-level (300/600 mg)	9.92	1	154	0.002
order* drug (y/n)	8.91	1	154	0.003
order*drug-level (300/600 mg)	0.21	1	154	0.646
drug (y/n) * drug-level (300/600 mg)	3.13	1	154	0.078
order* drug (y/n) * drug-level (300/600 mg)	0.53	1	154	0.465

**Fig. 4:**
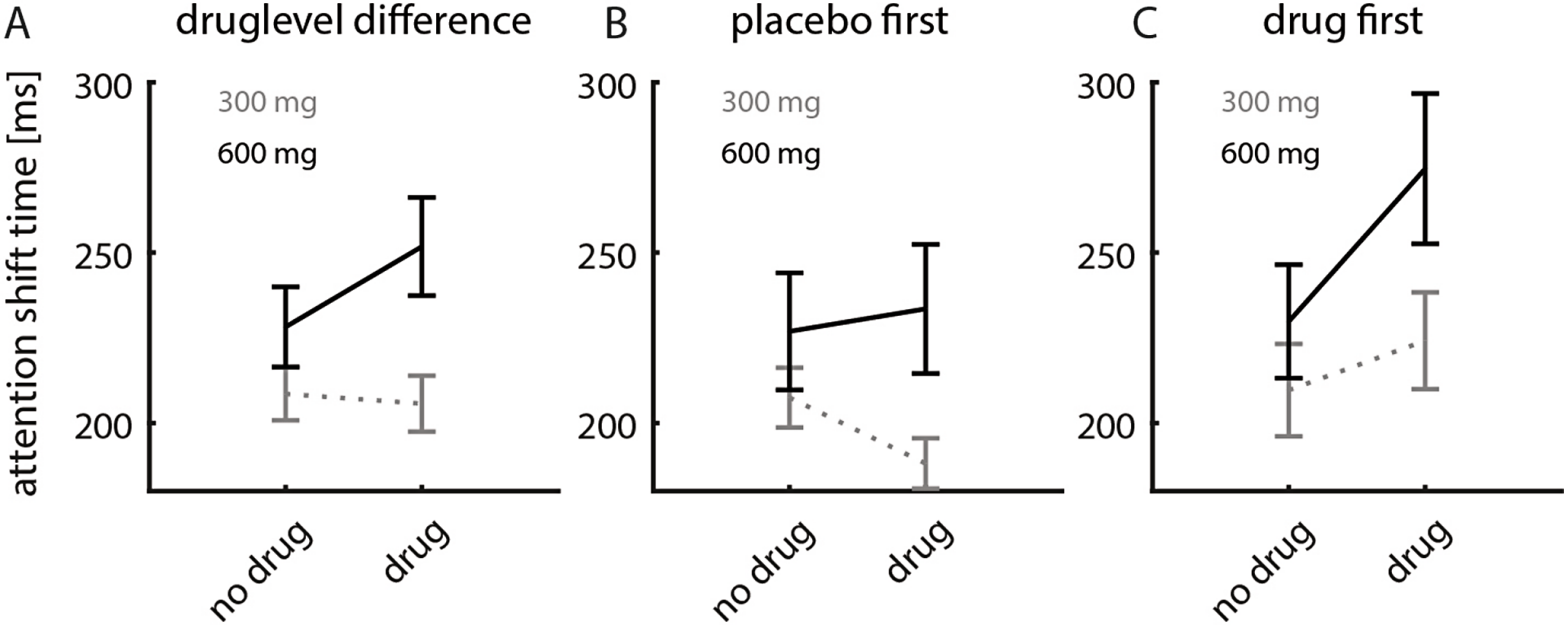
Average change in readout-delays for different drug level (300 mg vs. 600 mg). **A**) Difference in average readout-delays between drug and no drug conditions, independent of the order of drug/placebo session. **B**) Change in readout-delays for sessions where the placebo was taken in the first session. **C**) Change in readout-delays for sessions where the drug (scopolamine) was taken in the first session

Figure 3A shows that subjects’ readout-delays were longer when they were under the influence of scopolamine in the first session, compared to the second session (when they were not under the influence of scopolamine). Separating these effects for different drug levels shows this effect more clearly (Fig. 4)

Figure 4A shows that readout-delays were similar between no drug (placebo) and drug session when a dose of 300 mg was given in the drug session, (as reported above, the 300 mg dose did not affect readout-delays; main effect of drug: *p* = 0.689, F(1,234) = 0.2, mixed model ANOVA). When a 600 mg dose was given in the drug session readout-delays were increased compared to placebo session (main effect of drug: *p* = 0.004, F(1,228) = 8.7, mixed model ANOVA). Figure 4A thus highlights the drug-level effect previously described (see also Fig. 2; Table 1). When placebo was taken in the first session and 300 mg scopolamine were taken in the second session, subjects had shorter readout-delays in the second session (*p* = 0.002, sign rank test, Fig. 4B). No significant difference was found between sessions, when 300 mg scopolamine were taken in the first session (*p* = 0.057, sign rank test). Conversely, when 600 mg scopolamine were taken in the second session, no difference between sessions was found (*p* = 0.391, sign rank test). However, when 600 mg scopolamine were taken in the first session, readout-delays were significantly longer than in the second session (*p* = 0.006, sign rank test, Fig. 4B). This pattern might suggest that low dosages of scopolamine decrease readout-delays, while high dosages increase them. However, it becomes clear that this is not the case, when analysing the data from sessions where the drug was taken first (Fig. 4C). In this case drug sessions always resulted in longer readout-delays, when compared to placebo sessions. The data from Fig. 4B and C visually underpin the significant order* drug (y/n) interaction effect (Table 1). An order effect would demonstrate that subjects show some form of learning between sessions. While there was no main effect of order present, the interaction effect between order and drug (y/n) demonstrates that the learning (order effect) depends on whether scopolamine was taken in the first session. The design involved in the study described so far does not allow to determine the contribution of muscarinic receptors to learning directly, as performance in one of the two session was always affected by muscarinic blockade, in addition to the possible effect on consolidation that takes place between session.

We therefore performed an additional study (Study 2, methods), where performance within a session was always under control conditions (neither drug nor placebo was taken). The independent variable was whether subjects received scopolamine (group 1, *n* = 10, 2*300 mg Kwell pills) immediately after the first session (to potentially affect consolidation), or whether they received placebo after the first session (group 2, *n* = 10, 2 unflavoured glucose pills).

**Fig. 5:**
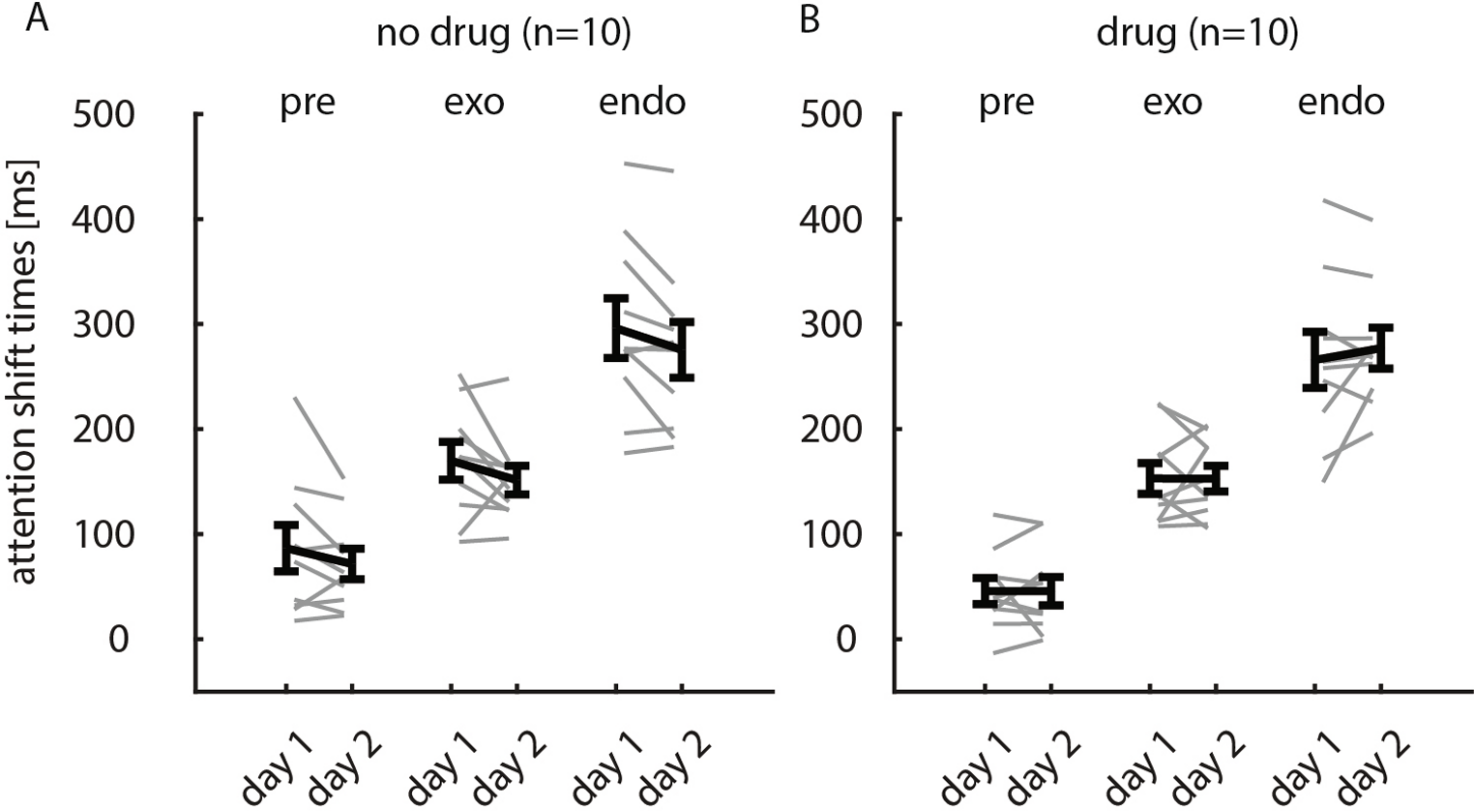
Average readout-delays (across the 3 conditions) as a function of session (day 1, day 2). **A**) Data from subjects who took placebo after the first session. **B**) Data from subjects who took the drug (600 mg scopolamine) after the first session. Mean readout-delays along with S.E.M are indicated along the y-axis in black, grey lines show means for individual subjects

Figure 5A shows that the average readout-delays were reduced in subjects who took the placebo pills after the first session for all 3 cue-types. This was not the case for the group of subjects who took scopolamine after the first session. To determine which factors significantly affected readout-delays we performed a mixed-model ANOVA (Table 3). Cue-type significantly affected performance, drug (y/n) had no effect on its own, there was a mild trend that day (session 1/2) influenced readout-delays, but critically there was a significant interaction between drug (y/n) and day (session 1/session 2). From Fig. 5 it might appear that the placebo cohort had longer readout delays during the first session (which could be due to random sampling), but which might result in the drug*day interaction seen. However, we did not find group differences on day 1 if tested across cue conditions (mixed-model ANOVA p_cue_<0.001, p_group_=0.067, p_group*cue_=0.822), or individually for the different cues (t-test, p_pre_=0.11, p_exo_=0.45, p_endo_=0.43).

**Table 3:** Mixed model ANOVA details relating to study 2. *Factor* indicates the parameter of interest, *df* shows the degrees of freedom, *F* and *p* give the F- and p-values respectively. * symbol denotes interaction between factors

Factor	df	F	*P*
Intercept (subject effects)	(1,108)	265.3	< 0.001
cue-type	(2,108)	182.1	< 0.001
drug (y/n)	(1,108)	0.8	0. 368
day	(1,108)	3.2	0.077
drug (y/n)* cue-type	(2,108)	1.5	0.232
day* cue-type	(1,108)	0.1	0.896
drug (y/n)*drug	(1,108)	7.2	0.009
day*drug (y/n)*cue-type	(2,462)	0.4	0.663

**Fig. 6:**
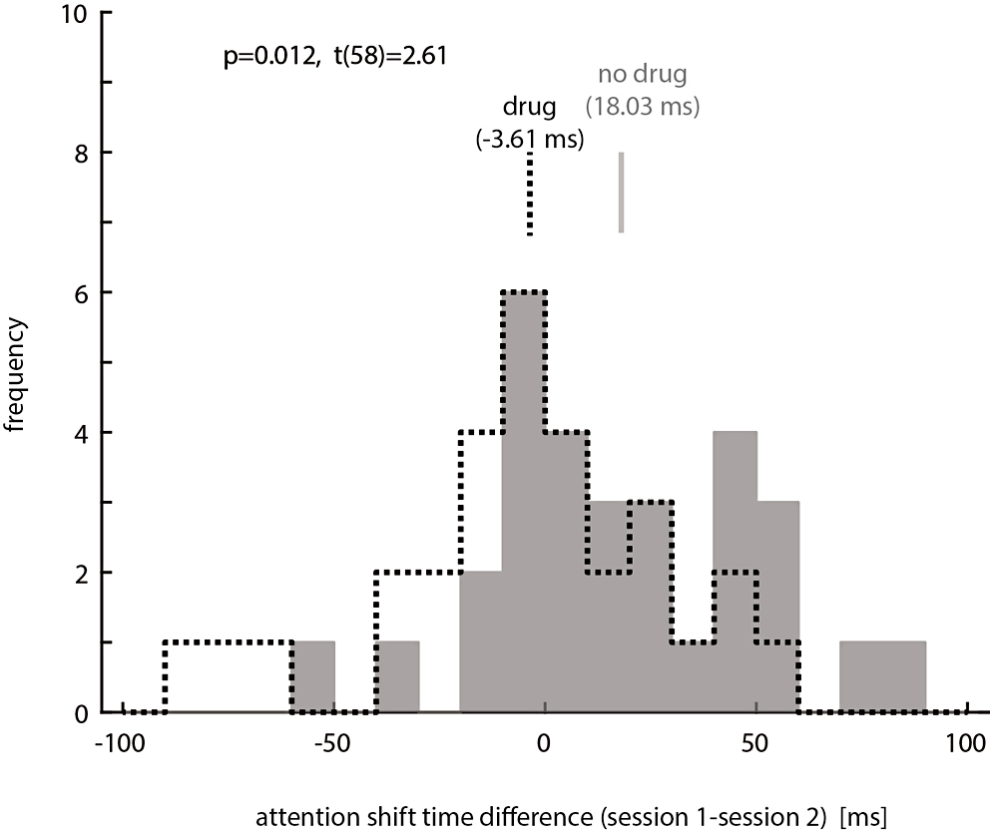
Readout-delay differences between sessions (readout-delay day 1 minus readout-delay day 2). Grey histogram shows the distribution for the control group. Dashed black line histogram shows the distribution for the drug treatment group. Vertical black and grey bars indicate group means. Positive values indicate that readout-delays were longer in the first session, negative values indicate that readout-delays were longer in the second session. P-value (along with t-statistic) at the top indicates that distributions were significantly different

To further investigate the interaction between drug (y/n) and day, we subtracted the readout-delays in session 2 from the readout-delays in session 1. This indicates whether a systematic change occurred between sessions, and whether this differed for the two treatment groups. The distribution of differences for the two groups is shown in Fig. 6.

The control group mostly had positive readout-delay differences, with a mean difference of 18.03 ms (Fig. 6). This indicates that readout-delays were affected by learning in this group (becoming faster). Conversely, the group who took scopolamine after the first session showed many negative readout-delay differences with a mean difference of (-3.61 ms), suggesting that in this group no learning took place and readout-delays did not become faster. The readout-delay differences between groups were significantly different (t(58), *p* = 0.012, t-test). Critically, the distribution of control subjects was significantly different from zero (*p* = 0.0039, t(29) = 3.14), while the distribution of subjects exposed to scopolamine was not (*p* = 0.551, t(29)=-0.603). Altogether this demonstrates that readout-delays were subject to learning (subjects get faster between sessions), but this was blocked when subjects were exposed to muscarinic blockade during the immediate consolidation period.

## Discussion

In line with previous reports (Carlson et al. 2006; Chakravarthi and VanRullen 2011) we found that readout-delays were fastest for the pre-cue conditions, followed by exogenous shift, and with endogenous shift being slowest. Muscarinic receptor blockade through oral scopolamine application affected how quickly subjects detected and reacted to relevant cues and read the time on the clock. Scopolamine increased readout-delays in a dose dependent manner. Muscarinic challenge mostly affected the pre-cuing condition, with smaller effects on exogenous cuing, and no significant effects on readout-delays in the endogenous cuing conditions. Additionally, muscarinic receptor blockade through oral scopolamine application immediately after session 1 reduced learning induced improvements of attention related read-out delays. Thus, muscarinic receptors are also involved in consolidation.

The contribution of cholinergic mechanisms to attention has been shown in many different settings (Nobili and Sannita 1997; Robbins 1997; Baxter and Chiba 1999; Davidson et al. 1999; Sarter et al. 1999; Bentley et al. 2003; Dalley et al. 2004; Herrero et al. 2008; Parikh and Sarter 2008; Thienel et al. 2009; Deco and Thiele 2011; Gratton et al. 2017; Dasilva et al. 2019), but has also often been disputed (e.g.Hangya et al. 2015). Attentional selection is assumed to be driven by frontal and parietal cortex, whereby the dorsal attention network is involved in top-down attention and the ventral network is involved in bottom-up selection (Corbetta and Shulman 2002). Parietal and frontal regions influence sensory processing directly via feedback (Moore and Armstrong 2003). How would the muscarinic signalling then be involved? Frontal areas connect to cholinergic neurons in the basal forebrain which in turn have ascending projections to sensory areas (Russchen et al. 1985; Sarter et al. 2005). If these ascending projections from the basal forebrain were relevant for attention induced improvements of sensory processing, then muscarinic blockade would dampen this (Herrero et al. 2008, 2017; Thiele and Bellgrove 2018). But frontal and parietal areas themselves receive cholinergic input, which is relevant for attentional performance (Sarter and Bruno 1997; Parikh et al. 2008; Parikh and Sarter 2008; Gritton et al. 2016; Sarter et al. 2016; Dasilva et al. 2019). Reduced muscarinic receptor availability could alter overall drive in those networks, hinder competitive interactions and thereby affect how task specific populations can assemble, or how they function (Thiele and Bellgrove 2018). Our data show that muscarinic receptor blockade had the largest effect on pre-cued readout-delays, which suggests that it reduces the ability to pre-allocate attention and maintain it at a peripheral location. The latter would hint at an impairment of spatial working memory and/or sustained spatial attention. Moreover, it increased readout-delays for exogenously cued targets. Interestingly, both the pre-cued conditions and exogenously cued conditions had a peripheral cue to indicate when the time on the clock should be read. Thus, the effect of muscarinic challenge on pre-cue conditions may be combination on reduced ability to pre-allocate attention and slowed peripheral cue detection (and subsequent attention shifting if not already pre-allocated), while for the exogenous cue condition it may be an effect on peripheral cue detection (and subsequent attention shifting) alone. The cholinergic system has been shown to be critically important in peripheral cue detection (Parikh et al. 2007), which is in line with our data. Critically, central (endogenous) cuing was unaffected by muscarinic challenge.

Muscarinic receptors also modulate visual perception (Zinke et al. 2006; Bhattacharyya et al. 2012; Pinto et al. 2013; Herrero et al. 2017) affecting contrast sensitivity and orientation selectivity in primary visual cortex. Our task involved a determination of the orientation of the clock hand. Impoverished orientation selectivity would mostly likely result in increased variance of the readout-delays, not a systematic increase. Reduced contrast sensitivity could in principle result in delayed read-out times, but this would affect all three cuing conditions equally, which is not what we found.

Our results of muscarinic receptor contribution to consolidation and learning aligns with studies showing a cholinergic role in neuronal plasticity (McKenna et al. 1989; Metherate and Weinberger 1990; Weinberger and Bakin 1998; Barros et al. 2002) and learning (McGurk et al. 1988, 1991; Carli et al. 1997; Izquierdo et al. 1998; Thiel et al. 2002; Hasselmo 2006; Barker and Warburton 2009; Rokem and Silver 2011; Beer et al. 2013; Thiele 2013; Chamoun et al. 2017). Studies investigating muscarinic involvement in neural plasticity often targeted effects in sensory areas (McKenna et al. 1989; Brocher et al. 1992; Hasselmo and Barkai 1995; Kilgard and Merzenich 1998; Ego-Stengel et al. 2001; Weinberger 2004; Miasnikov et al. 2008). The effects seen under these conditions were not dissimilar to what occurs during perceptual learning (Karni and Sagi 1991; Schoups et al. 1995, 2001; Gold et al. 1999; Pleger et al. 2003; Li et al. 2005; Sanayei et al. 2018), an implicit type of memory. Other types of implicit learning, such as conditioned avoidance, cue triggered reward, and motor learning, depend at least partly on cholinergic signalling and plasticity of the basal ganglia (Packard and Knowlton 2002; Ostlund et al. 2017). Whether the learning seen in our study is related to perceptual, cognitive, or covert motor learning is unclear. Whatever the source, its consolidation is dependent on muscarine receptor availability.

### Conclusions

We found that muscarinic blockade affected readout delays in a dose dependent manner. These effects were mostly present for pre-cue conditions, followed by exogenous attention shifting conditions, while no effects were found for endogenous attention shifts. It suggests that muscarinic blockade interferes with the ability to pre-allocate attention, and with the speed to use peripheral cues for read-out of information. Moreover, application of the muscarinic blocker scopolamine during the immediate consolidation period resulted in reduced improvements of attention read-out delays.

## Electronic supplementary material

Below is the link to the electronic supplementary material.

Supplementary Material 1

## Data Availability

Data are available upon request.
