# Supplementary material for: Effect of muscarinic blockade on the speed of attention shifting, read-out delays and learning
Source: Psychopharmacology (Berl). 2025 Feb 15;242(8):1757–68. doi: 10.1007/s00213-025-06757-3 (PMC12296772; doi:10.1007/s00213-025-06757-3)
Supplement: Supplementary file 1 — Supplementary Material 1 [file 213_2025_6757_MOESM1_ESM.docx]

# Supplementary Materials

**Effect of muscarinic blockade on the speed of attention shifting and learning**

Alexander Thiele^1^, Agnes McDonald Milner^2^, Corwyn Hall^2^, Lucy Mayhew^2^, Anthony Carter^2^, Sidharth Sanjeev^2^

^1^Bioscience Institute of Neuroscience, Newcastle University, Framlington Place, Newcastle upon Tyne, UK, NE2 4HH

^2^School of Psychology, Newcastle University, Framlington Place, Newcastle upon Tyne, UK, NE2 4HH

# Exclusion criteria based on scopolamine medication counter-indications:

Subjects were excluded if they had any of the of the following conditions:

- Glaucoma.
- Blockage of intestines (Paralytic ileus).
- Narrowing of the stomach outlet (Pyloric stenosis).
- Myasthenia gravis.
- Enlarged prostate gland.
- were under medical care, especially for heart, metabolic,
- gastrointestinal, liver or kidney conditions.
- Had previously had a sudden painful inability to pass urine.
- Had ulcerative colitis.
- Had diarrhoea or fever.
- Had Down’s Syndrome.
- Suffered from seizures or fits.

Or took any of the following medicines,

- Amantadine (an antiviral).
- Antihistamines.
- Antipsychotics.
- Antidepressants.
- Linezolid (and antibiotic).
- Domperidone and metoclopramide (for nausea and vomiting).
- Sublingual nitrates (for angina).
